# Immunomodulatory effects of chitosan oligosaccharides produced by chitosanase from *Bacillus* isolate

**DOI:** 10.1186/s13568-025-01958-7

**Published:** 2025-10-13

**Authors:** Akram N. Salah, Nooran S. Elleboudy, Mohamed M. S. Farag, Talat A. El-Kersh, Mahmoud A. Yassien

**Affiliations:** 1 Experimental and Advanced Pharmaceutical Research Unit, Faculty of Pharmacy, Ain Shams University, Organization of African Unity St., Abbassia, Cairo 11566 Egypt; 2 Department of Microbiology and Immunology, Faculty of Pharmacy, Ain Shams University, Organization of African Unity St., Abbassia, Cairo 11566 Egypt; 3 Botany and Microbiology Department, Faculty of Science, Al-Azhar University, Cairo, 11884 Egypt; 4 Biomedical Research Department, Armed Forces College of Medicine (AFCM), Cairo, Egypt; 5 The Regional Center for Mycology and Biotechnology, Al-Azhar University, Cairo, Egypt; 6 Microbiology and Immunology Department, Faculty of Pharmacy, Badr University in Cairo (BUC), Badr City, 11829 Cairo Egypt

**Keywords:** Chitosan oligosaccharides, Chitosanase, *Bacillus cereus*, Immunomodulatory, Anti-inflammatory, Immunostimulatory, Lc–ms

## Abstract

Chitosan oligosaccharides (COSs) are short-length oligomers produced by the action of chitosanase enzymes. These oligomers are characterized by high water solubility and bioavailability. COSs have demonstrated several biological activities in the medical field, including antitumor, antimicrobial, antioxidant, and immunomodulatory. This study aimed to assess and purify bio-produced COSs obtained from a culture containing a locally isolated *Bacillus cereus* strain from an Egyptian soil sample and investigate their biological activities, such as immunostimulatory and anti-inflammatory activities. The COSs were bio-produced by fermentation of a native *B. cereus* strain in media containing colloidal chitosan hydrolyzed by the action of chitosanase enzyme. COSs were then purified on a column packed with a Sephadex LH-20 column. The purified form of COSs were quantified and detected according to their molecular weights and degree of polymerization by UPLC-Mass spectroscopy. The purified COSs were detected as polymers of GlcN at different degrees of polymerization (DP1-DP6) and different molecular weight ranges (m z^−1^). The purified COSs were administered orally to groups of mice to evaluate their immunostimulatory effects on cyclophosphamide-induced immunosuppression by measuring cytokine levels in their thymus glands and spleens. Cytokine analysis revealed that COSs enhanced the release of IL-6, TNF-α, and IFN-γ in both the thymus and spleen (*p* < 0.05). The activity of COSs as an anti-inflammatory was assessed by inducing edema in the paws using carrageenan. COSs’ dose of 500 mg kg^−1^ exhibited a potential anti-inflammatory effect when compared to dexamethasone (*p* < 0.05). This study concluded that the bio-produced COSs have considerable potential and are promising as a drug with immunostimulant activities. These observed activities resulted from the ability of COSs to stimulate the production of different cytokine markers in safe doses. Also, these COSs showed significant anti-inflammatory activity when administered orally.

## Introduction

Chitooligosaccharides (COSs) are oligomers that are obtained from the chitosanase enzyme hydrolysis of the -b-1,4-glucosidic bond between the D-glucosamines, GlcNs in chitosan polymer (Lourenço et al. 2023). These COSs are characterized by short lengths of oligomers with a wide range of molecular weights (Patra et al. 2022), water solubility (Fawzya et al. 2018), and low viscosity, which enhances their biological activities (Jitprasertwong et al. 2021).

Chitooligosaccharides can be prepared by chemical or enzymatic hydrolysis of chitosan. The chemical preparation of COSs can be carried out by oxidation reactions using strong oxidizing agents such as hydrogen peroxide (H_2_O_2_) to convert chitosan into COSs in the presence of acid (Joshi et al. 2022). While the enzymatic preparation of COSs can be performed by the action of chitosanase enzyme, which is characterized by high specificity to chitosan, making it well-suited for chitosan degradation to yield COSs precisely (Hao et al. 2022).

Enzymatic hydrolysis of chitosan has been made by the action of bacterial chitosanase, which is biologically produced from bacterial strains (e.g., *Bacillus* spp., *Pseudomonas* spp., and *Streptomyces* spp.) in a culture medium containing chitosan as a carbon source. In microbial culture supernatants, a large amount of COS can be produced as a result of the -b-1,4-glycosidic linkages cleavage in chitosan, and each molecule of chitosanase could release the number of micromoles of COSs per minute that can be purified and quantified by different methods such as size exclusion chromatography and mass spectroscopy (Choi et al. 2004).

Microbial production of chitosanase is widely used as a suitable method for large-scale production of COSs by enzymatic hydrolysis, which has the privilege of low operation cost, reduced production time, and without any environmental damage (Xia et al. 2023).

These COSs have demonstrated several biological activities in the medical field, including antitumor by their cytotoxic effect against different tumor cells (Wu et al. 2019), antioxidant activities by their ability to diminish 2,2-Diphenyl-1-picrylhydrazyl (DPPH) radicals (Marmouzi et al. 2019; Yu et al. 2023), and rejuvenating different antioxidative enzymes (e.g., catalase and superoxide dismutase), and glutathione impaired by different oxidative stressors (Liu et al. 2022; Cheng et al. 2024).

Also, COSs showed immunomodulatory effects by inducing the cytokines’ production, such as IFN-γ, IL-1, 6, 10, and TNF-α, and antimicrobial effects against Gram-positive and Gram-negative bacteria and fungi that are mainly applied in medicine and pharmaceutics (de Andrade et al. 2021; Fang et al. 2024).

*Bacillus* spp. are presented and reported for the fermented and enzymatic production of COSs, with the advantage of bio-producing COSs in a purified form with low cost (Ling et al. 2022).

In the present study, a locally isolated and native *B. cereus* strain was selected to produce COSs by its hydrolysis of chitosan with chitosanase. The produced COSs were purified and used to investigate their biological activities, such as immunostimulatory and anti-inflammatory activity.

## Materials and methods

### Microorganism isolation and identification

A high chitosanase-producing bacterial strain (lab-coded with SE42) was locally isolated from a soil sample in Egypt and then identified using 16S rRNA gene sequencing. It was deposited in NCBI GenBank with accession no. PP093334.1 (www.ncbi.nlm.nih.gov). The CLUSTAL X (Conway Institute, UCD Dublin, Dublin, Ireland) was used for sequence alignment, and MEGA 11.0 (Mueller Laboratory, Pennsylvania) for the phylogenetic tree using the neighbor joining (NJ) construction method. This analysis involved 23 nucleotide sequences. All ambiguous positions were removed for each sequence pair using the pairwise deletion option. There were a total of 1543 positions in the final dataset with a bootstrap value of 1000 replicates. Also, the SE42 strain was added to the World Data Centre for Microorganisms Culture Collection at Ain Shams University (CCASU).

### Production of COSs

For bioproduction of COSs, 5 ml of bacterial culture of the tested isolate (with a count 1.5 × 10^8^ CFU ml^−1^) was added to 100 ml of the chitosanase detecting broth (each 1 L of the medium contains 10 g colloidal chitosan solution, 0.5 g NaCl, 5 g yeast extract, 0.5 M KCl, 1 g NH_4_Cl, 0.24 g MgSO_4_, 0.7 g NaH_2_PO_4_, 0.34 g Na_2_HPO_4_, 0.1 g CaCl_2_) in 500-ml Erlenmeyer shake flasks (120 rpm) and incubated at 30 °C for 48 h. The final pH of the medium was 6.5–7 (Choi et al. 2004).

The colloidal chitosan was purchased from Sigma Aldrich Company (LLC, Taufkirchen, Germany), the colloidal solution of chitosan was specifically used because of its high solubility to be widely used in COSs production (Chen et al. 2022), it was prepared by dissolving 10 g of powdered chitosan in 400 ml of distilled water by stirring while adding 90 ml 1 M 2% (v/v) acetic acid, completing to 1 L with water for 6–8 h until the chitosan has completely dissolved, filtering insoluble chitosan using sintered glass. The produced COSs were quantitatively evaluated by dinitrosalicylic acid assay (Miller 1959).

### Chitosanase activity assay

The dinitrosalicylic acid (DNS) method was carried out as described by Miller (1959) and modified by Zhang et al. (2021), with 1% (w/v) colloidal chitosan. The substrate and D-glucosamines (Sigma Aldrich, Co. LLC, Taufkirchen, Germany) as the standard, 0.9 ml of 1% colloidal chitosan solution (dissolved in a 0.2 M sodium acetate buffer to pH 7) was combined with 0.1 ml of cell-free supernatant and the mixture was then incubated for 10 min at 37 °C before the reaction was terminated by adding 1 ml of DNS reagent (5 g of 3,5 dinitrosalicylic acid, 1 g of Phenol, 0.25 g of sodium sulfite, 5 g sodium hydroxide and dissolved in distilled water to 500 ml).

The resulting mixture was then centrifuged at 6000 × g rpm for 10 min to remove leftover chitosan. Subsequently, the mixture was boiled for 5 min, and the absorbance at 540 nm was measured. The definition of one unit (U) of chitosanase activity is the amount of enzyme needed to generate 1 µmol of D-glucosamine per minute under the specified conditions.

### Purification, detection, and analysis of COSs

After the fermentation process, the microbial cells were harvested by centrifugation (6500 × g) at 4 °C for 20 min, and the collected supernatant was used for the recovery of COSs.

The COS mixture obtained for purification using size exclusion chromatography (SEC) was loaded on a column (3 × 50 cm) packed with Sephadex LH-20 (Cytiva AB, Sweden) equilibrated with the sodium phosphate buffer (pH = 7.0). The supernatant flow rate on the column was 0.2 ml min^−1^. The collection frequency was once every 10 min.

The quantification of yield and detection of the purified COSs was achieved by running on ACQUITY UPLC–BEH, with column C18 1.7 µm–2.1 × 50 mm (Li et al. 2016) to detect different oligomers of COSs according to their retention times.

Detection and quantification of the molecular weight of COSs mixture was carried out by Mass spectroscopy (Waters Corporation, Milford, U.S.A) with the development of electrospray ionization (ESI) (Wang et al. 2024). They were connected to COSs’ molecular weights represented in m z^−1^ values on the mass spectroscopy chart. COSs provided oligomers with different degrees of polymerization (DP), which were also reported.

The bio-produced COSs mixture was freeze-dried using Lab − 60 ℃ Vacuum Freeze Dryer Lyophilizer (Avenue, Watertown, MA 02472) and stored in capped aliquot tubes (each tube contains 1 gm) at 4 °C to be used for further studies.

### Animal experiments to assess immunomodulatory effects

All these in vivo experiments were carried out in the animal house at the Faculty of Pharmacy, Ain Shams University, Cairo, Egypt. Ethical approval for the study was granted by the Faculty’s Ethical Committee (Approval No. REC-289).

The animals used were Balb/c male mice with a mean weight of 24 g (range from 21 to 39 g), and each group consisted of 8 mice. Mice were kept in boxes and a controlled environment at 25 ± 2 °C, and a relative humidity of 40–65% was achieved with a twelve-hour dark cycle.

#### Animals

The animals were divided into 5 groups; group I received normal saline solution considered as a negative control, while group II received Cyclophosphamide (60 mg kg^−1^) (Baxter Oncology GmbH, Germany) as a positive control, mice groups III, IV, and V get COSs with three concentrations (1000, 500, and 250 mg kg^−1^, respectively).

Four groups (II, III, IV, and V) of mice were treated with Cyclophosphamide (Baxter Oncology GmbH, Germany) with a dose of 60 mg kg^−1^day^−1^ from 21 to 30 days. The mice were weighed, and the cervical dislocation was carried out for euthanizing after the last medication injection within 24 h. Immunological organs (spleens and thymus glands) indices were assessed.

#### Acute toxicity assessment and screening

Four concentrations of COSs (1000, 500, 250, 100, 75, and 50 mg kg^−1^) were given orally to four groups of mice, using a saline solution as a solvent. Mice were continually monitored for 4 h to identify any alterations in their behavioral or autonomic reactions, including alertness, irritability, spontaneous activity, corneal reflex, pinna reflex, salivation, urine, and piloerection (Narayanan et al. 1999). The mortality rates throughout the experimental period (2 weeks) were documented. According to the results obtained, the dosages that showed safety were selected to investigate the COSs’ anti-inflammatory effects.

#### Thymus glands and spleen indices

The spleen and thymus gland of each mouse were aseptically extracted and weighed in each mouse group, and their indices were computed as follows:

$$ \begin{aligned} {\text{Thymus}}/{\text{spleen}}\;{\text{index}} & = {\text{The}}\;{\text{average}}\;{\text{weight}}\;{\text{of}}\;{\text{the thymus}}/{\text{spleen}} \\ & \quad {\text{of}}\;{\text{the}}\;{\text{mice}}\;{\text{group}}\;{\text{body}}\;{\text{weight}}\;{\text{of}}\;{\text{the}}\;{\text{group}} \\ \end{aligned} $$

#### Immunostimulatory assay

The excised spleens and thymus glands homogenates in 0.2 g ml^−1^ cold physiological saline were prepared and then centrifuged at 3000 × g for 10 min at a cooling temperature (4 ℃) to obtain supernatant. The cytokines (IL-6, TNF-α, and INF-γ) were assayed by ELISA using specific kits (Mei et al. 2013).

### Anti-inflammatory assay

#### Animals

Seven groups of Balb/c male mice. Mice were kept fast for 24 h before carrying out the experiment, and only water was given when necessary.

#### Acute inflammation measurement

The anti-inflammatory activity of the tested COSs was studied by inducing edema in mice’s paws using carrageenan; this method was described previously by Winter et al. (1962). The selected doses of COSs were administered 60 min before carrageenan injection.

The groups of mice were divided as; group I received normal saline solution (Otsuka Egypt, Giza, Egypt) considered as a negative control, while group II received Dexamethasone (Sigma for pharmaceuticals, Cairo, Egypt) as corticosteroid drugs positive control with a dose 1 mg kg^−1^, group III received Indomethacin (MUP, Cairo Egypt) with a dose 10 mg kg^−1^ as a non-steroidal anti-inflammatory drug to confirm the validity of the experiment and ensure the carrageenan validity for inducing paw edema in this experiment, groups IV, V, VI, and VII were administrated with an oral dose of COSs with concentrations of 50, 100, 500 and 1000 mg kg^−1^ (Marshall et al. 1989).

The induction of edema in this experiment was performed by injecting 0.015 ml of 2% carrageenan into the right hind paw’s plantar surface. The paw thickness difference of the right hind paw was quantified to indicate edema formation and was measured in centimeters using a digital caliper (Skylake Medical Co., China) at 0, 0.5, 1, 2, 4, 8, 24, and 48 h.

### Statistical analysis

All data from experiments were expressed as mean ± standard deviation (SD). The mean value differences between groups were evaluated using a two-way analysis of variance (ANOVA), Bonferroni-Holm post-test, which is a post hoc test used to compare the studied groups with each other’s and the Kruskal–Wallis non-parametric test, which was used instead of ANOVA if there is no normally distributed data in some groups, employing GraphPad Prism software v.8.0.1. The level of significance was attributed to differences with *p* value < 0.05, while differences with *p* value < 0.01 were regarded as highly significant.

## Results

### Bacterial strain identification

The preliminary and biochemical identifications showed that the bacterial strain was Gram-positive bacilli with a spore formation, motile, aerobic, catalase positive, non-crystal forming, starch and casein hydrolysis positive.

**Fig. 1:**
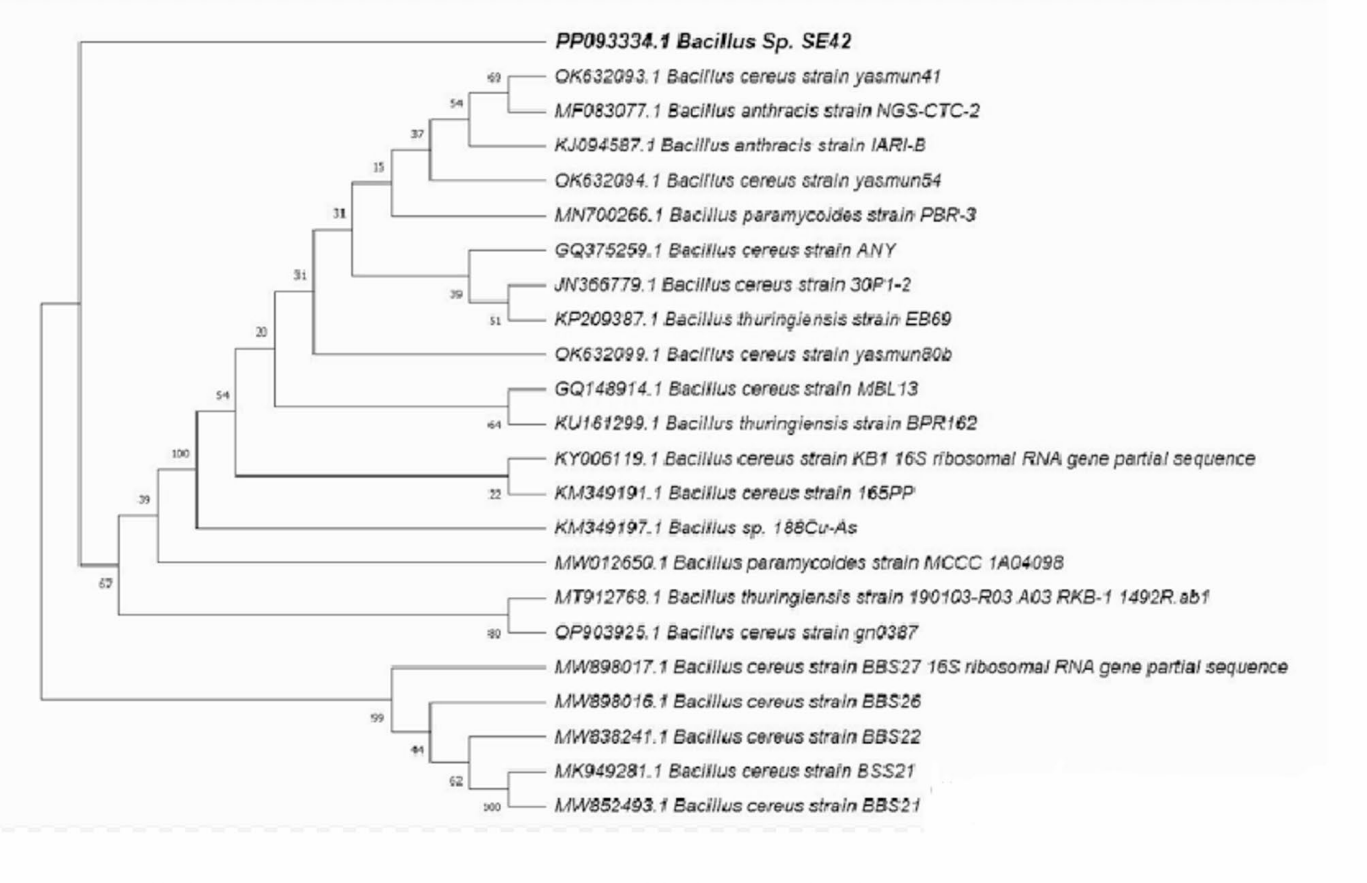
The strain SE42’s phylogenetic tree with bootstrap values. The MEGA 11.0 software was utilized to make phylogenetic analysis using the neighbor-joining (NJ) construction method

The strain SE42 was analyzed for molecular identification by cloning and sequencing a 1512 bp fragment of its 16S rDNA gene, which was assigned the GenBank accession number PP093334.1. The 16S rDNA gene sequence analysis showed a 99% similarity between the isolated strain (Lab-coded: SE42) and the *Bacillus cereus* strain based on the phylogenetic position of its 16S rDNA and bootstrap values, which equals 80% to *B. cereus* strain gn0387 and 100% to *B. cereus* strain BBS21 (Fig. 1).

Also, the SE42 strain was added to CCASU with strain code CCASU-2024-71.

### Production, purification, and analysis of COSs

After 48 h of *B. cereus* growth in culture medium, the reported culture conditions improved the chitosanase activity to produce COSs with a maximum of 1190 ± 20.1 U L^−1^ with a total yield of 2.16 g.

**Fig. 2:**
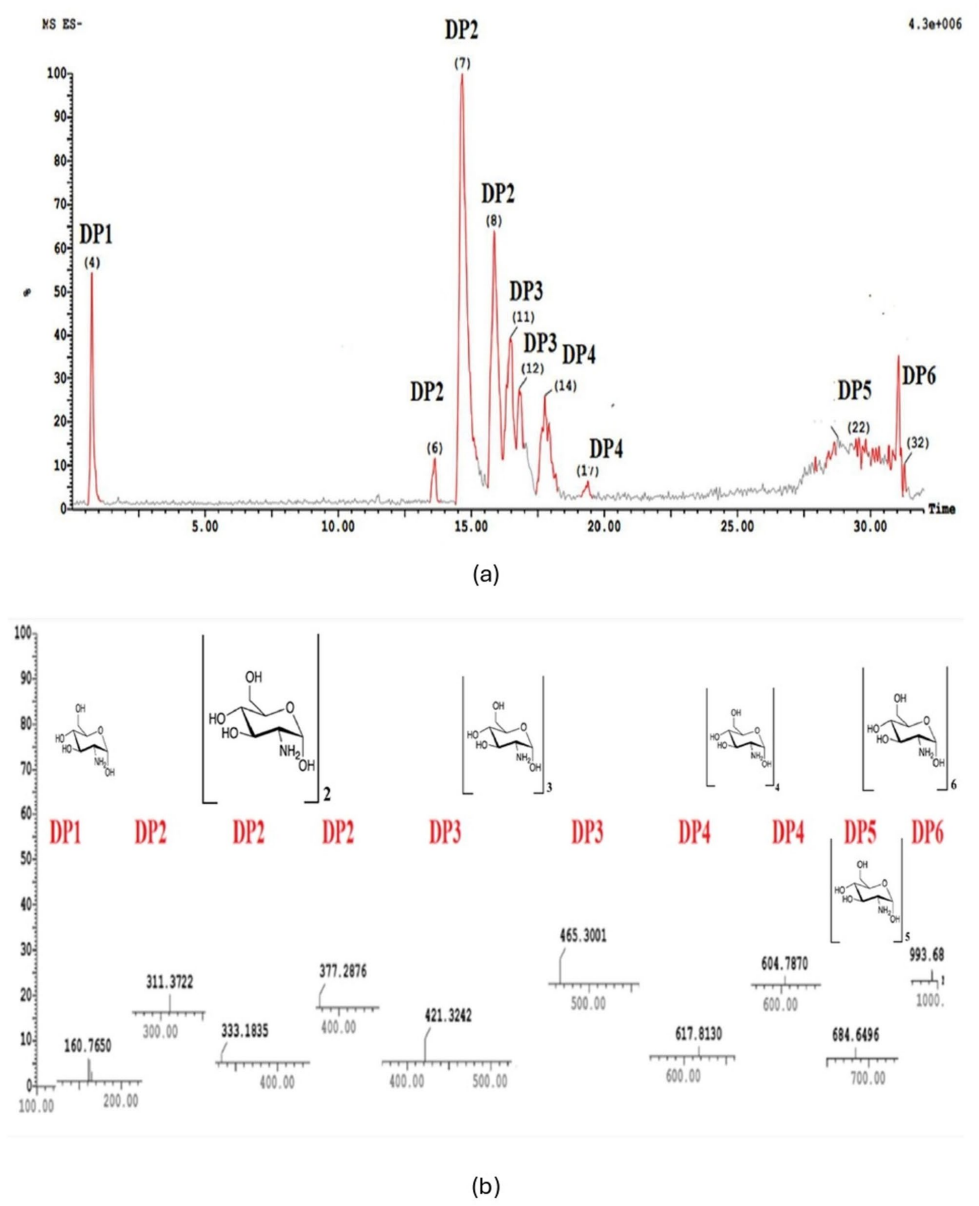
**a** The detection and quantification assay; UPLC chromatogram from *B. cereus* broth culture supernatant and COSs, showing a detectable degree of polymerization (DP) at different retention times. **b** The analytical and quantification method; LC mass spectroscopy of COSs mixture obtained from *B. cereus* culture supernatant detected fragment ions with different m z^−1^ values and different DP values (DP1-DP6). The rest of the mass fragment ions are represented in Supplementary files (S1)

The lc–ms spectrum is a sensitive and specific analytical method for detecting COSs. The full mass spectra of the oligomers of COSs were visualized at different retention times, and all COSs mixtures’ oligomers have been detected after 60 min of size exclusion column chromatography elution (Fig. 2a). The oligomers were detected as polymers of GlcN at different degrees of polymerization (DP). They were expressed as DP1-DP6. Other mass spectra of the COSs mixture are illustrated in Supplementary File 1 (S1).

The UPLC-mass spectroscopy of the COSs mixture obtained from *B. cereus* culture supernatant detected fragment ions. The lc mass fragment ions were visualized at different molecular weight ranges from 160.7. to 993.6896 m z^−1^ (Fig. 2b). Each peak represents a different chitooligomer with a definite DP. This COSs mixture was used in different doses in the following experiments.

#### Immunostimulatory

Different doses of COSs mixture that have different oligomers have been used in this study with different molecular weights (from 160.7 to 993.6896 m z^−1^) and different DPs (1–6). This COSs mixture was used for immunostimulatory and anti-inflammatory experiments.

#### The thymus gland and spleen indices measurement

**Fig. 3:**
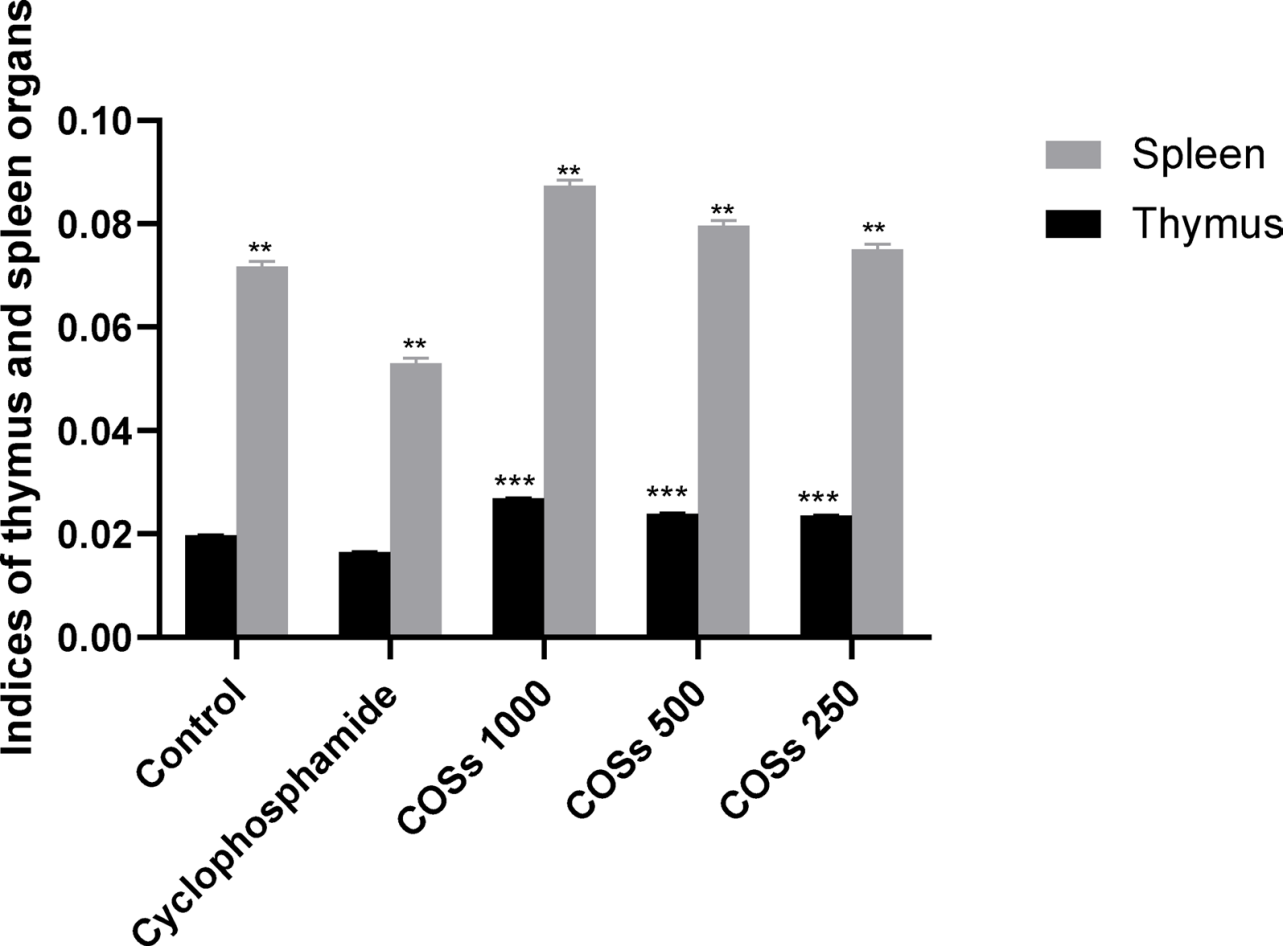
The immunostimulatory effect of COSs on immunocompromised mice showed an increase in thymus gland and spleen indices. The effect of COSs’ different concentrations on the spleen and thymus indices among cyclophosphamide-treated mice, ** there is a significant difference between groups and control (*p* < 0.0001), *** there is a significant difference between COSs-treated groups and the Cyclophosphamide group (*p* < 0.001)

As compared to the negative control group (normal saline), treatment with Cyclophosphamide made a significant decrease (*p* < 0.0001) in thymus glands and spleen indices. All the groups treated with COSs showed a significant increase (*p* < 0.001) in thymus glands and spleens’ indices, and their effect was concentration-dependent (Fig. 3).

#### The effect of COSs on cytokines among cyclophosphamide-treated mice

The groups treated with COSs showed a significant (*p* < 0.01) increase in the levels of IL-6 and INF-γ as compared to those of the control (cyclophosphamide-treated group). Regarding the level of TNF-α, a significant increase (*p* < 0.01) in its level was obtained in the thymus specimens after treatment with 1000 and 500 µg kg^−1^ (Table 1). On the other hand, the level of TNF-α in the spleen specimens was significantly (*p* < 0.01) increased only after treatment with 1000 µg kg^−1^ (Table 2).

**Table 1:** The effect of COSs’ different concentrations on the IL-6, TNF-α, and INF-γ levels in thymus samples

Groups	IL-6	TNF-α	INF-γ
Control	244.4 ± 7.64	211.17 ± 11.9	21.6 ± 2.06
Cyclophosphamide (60 mg kg^−1^ body weight)	217.93 ± 3.65a	191.8 ± 1.64a	17.77 ± 1.33a
COSs 1000 µg kg^−1^	486.93 ± 13.48a,c	346.33 ± 19.63a,d,f	39.07 ± 1.46a,c,f
COSs 500 µg kg^−1^	402.4 ± 13.04a,c,e,f	297.97 ± 15.06a,d	31.13 ± 1.82a,c,e
COSs 250 µg kg^−1^	355.433 ± 11.86a,c,e	251 ± 15.45a	27.07 ± 1.16a,c

a comparing with control at *p* < 0.01, b comparing with control at *p* < 0.05, c comparing with Cyclophosphamide treated group *p* < 0.01, d comparing with Cyclophosphamide treated group *p* < 0.05, e comparing with COSs (1000 µg ml^−1^) group *p* < 0.01, and f comparing with COSs (250 µg ml^−1^) group *p* < 0.05

**Table 2:** The effect of COSs’ different concentrations on the IL-6, TNF-α, and INF-γ levels in spleen samples

	IL-6	TNF-α	INF-γ
Control	339.13 ± 8.26	286.53 ± 11.34	14.57 ± 0.99
Cyclophosphamide (60 mg kg^−1^ body weight)	292.9 ± 10.11	218.81 ± 5.16b	12.93 ± 1.53
COSs 1000 µg kg^−1^	876.5 ± 26.68a,c	278.57 ± 3.49a,d	43.2 ± 2.49a,d
COSs 500 µg kg^−1^	593.93 ± 23.45a,c,e,f	235.2 ± 7.37a,e	31.07 ± 1.91a,d,e,f
COSs 250 µg kg^−1^	454.93 ± 6.54a,c,e	222 ± 16.71a	21.27 ± 1.94a,d,e

a comparing with control at *p* < 0.01, b comparing with control at *p* < 0.05, c comparing with Cyclophosphamide treated group *p* < 0.01, d comparing with Cyclophosphamide treated group *p* < 0.05, e comparing with COSs (1000 µg ml^−1^) group *p* < 0.01, and f comparing with COSs (250 µg ml^−1^) group *p* < 0.05

### Anti-inflammatory

The concentrations of COSs 50–1000 mg kg^−1^ did not show any symptoms of irritation, behavioral changes, or toxicity among the tested mice in this experiment.

All the indomethacin, dexamethasone, and COSs-treated groups showed a marked decrease in the thickness of the mice’s paws after edema induction.

**Fig. 4:**
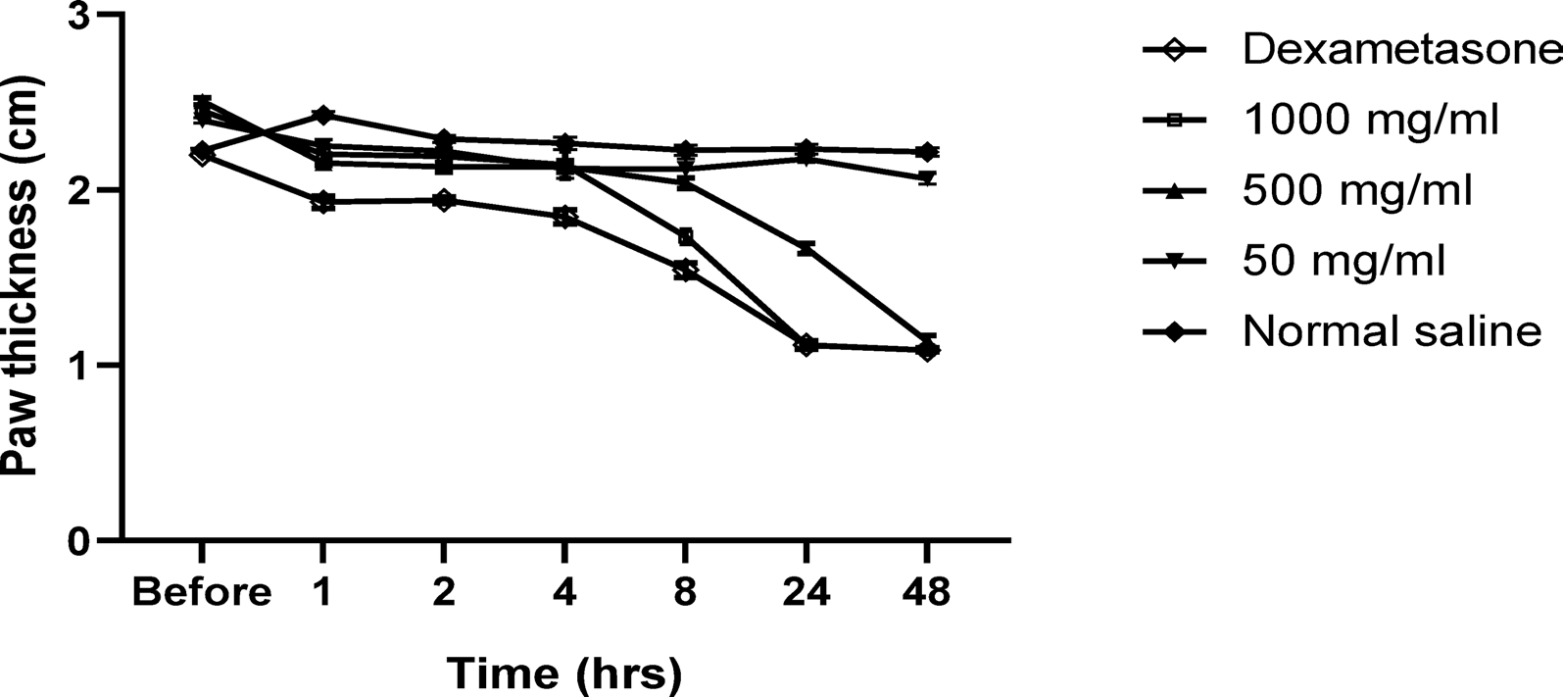
The anti-inflammatory effect of COSs is represented as a reduction of paw edema induced by carrageenan. The effect of three COSs doses (1000 and 500 mg kg^−1^) administered orally and positive and negative controls on the paw edema thickness (Mean ± S.D) (cm) along the time (h)

After one-hour edema induction, dexamethasone and COSs (1000 and 500 mg kg^−1^) treated groups showed a reduction in the paw thickness of 17.3%, 14.2%, and 8.5%, respectively. After 24 h of edema reduction, the percentage of reduction in the paw thickness increased to 52.1%, 54.3% and 32.8%, respectively. Regarding the COSs 100 mg kg^−1^ treated group, the reduction in edema thickness (10.6%) was observed only after 24 h of edema induction (Fig. 4).

**Fig. 5:**
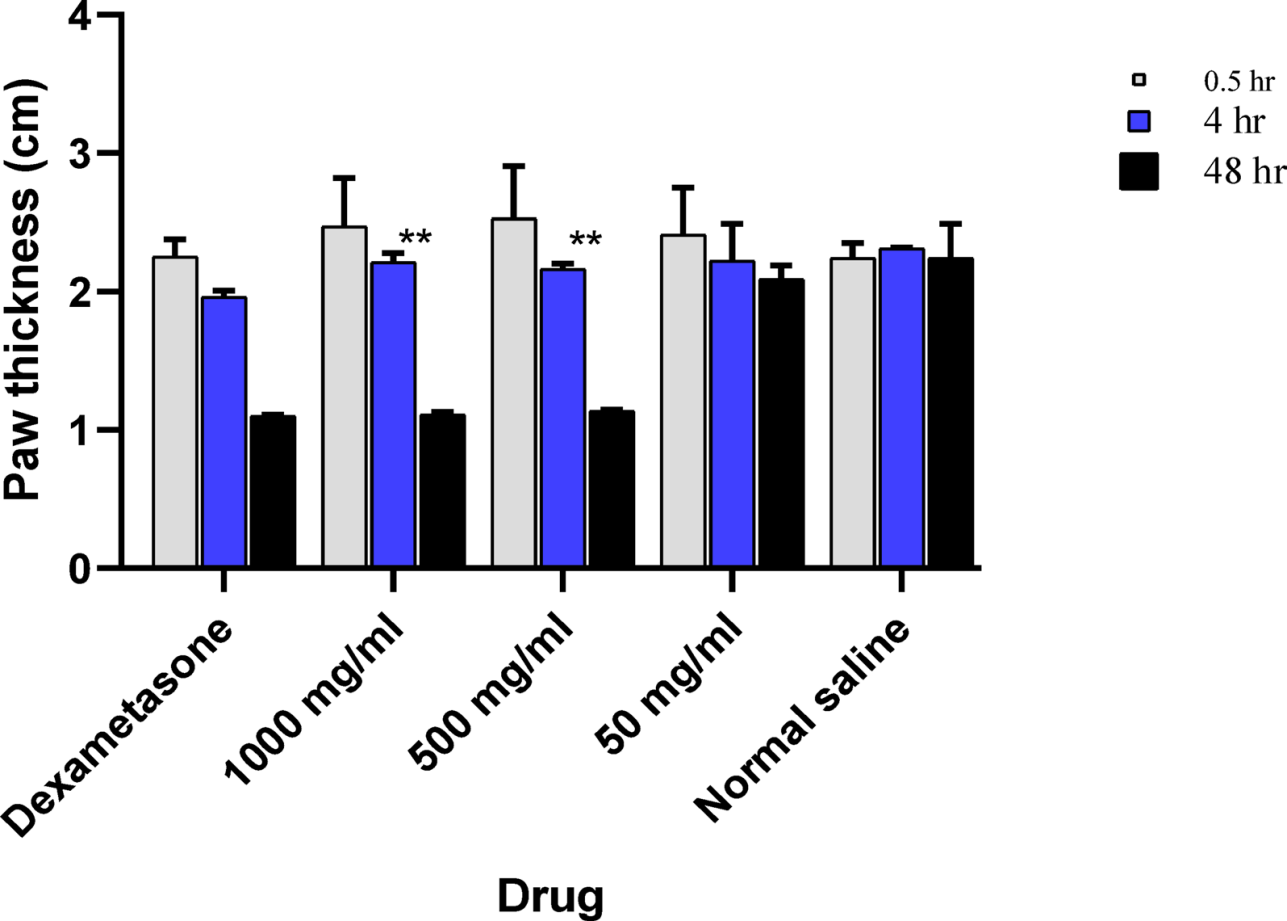
Different anti-inflammatory drugs were given to reduce the paw’s edema, which was induced by carrageenan. The effect of three COSs doses, dexamethasone, and normal saline on the paw edema of mice at three time points (0.5, 4, and 48 h)

For evaluation of the delayed anti-inflammatory effect of the tested COSs concentrations and the positive control (Dexamethasone), the paw’s thickness of their groups was measured at 0.5, 4, and 48 h after edema induction. According to the statistical analysis results of each group, a significant decrease (*p* < 0.001) in paw thickness was observed during the experiment period. However, no significant change in the paw thickness was observed in the negative control (normal saline) group (*p* = 0.28) (Fig. 5).

Regarding the COSs-treated groups, there is no significant difference between all COS-treated groups after 0.5 and 48 h (*p* > 0.05); however, after 4 h, the decrease in the paw thickness in the 50 mg kg^−1^ COSs group was significantly lower than that of the COSs treated groups (1000 and 500 mg kg^−1^). In comparison between dexamethasone and COSs-treated groups (1000 and 500 mg kg^−1^), no significant difference (*p* > 0.05) in the reduction of paw thickness after 4 and 48 h of edema induction. However, the dexamethasone-treated group showed a significant reduction (*p* < 0.05) in the paw thickness as compared to all the COSs-treated groups after 0.5 h of edema induction (Fig. 5).

## Discussion

The enzymatic hydrolysis of chitosan, giving pure chitooligosaccharides (COSs) products with different molecular weights, is widely used for different biological activities in pharmaceutical and medical fields, such as immunostimulatory as well as anti-inflammatory activities (Fang et al. 2024).

A locally isolated strain showed promising activity for the production of COSs after growth in chitosanase-detecting broth medium. The results of biochemical tests and genetic identification revealed that the isolate is identified as *Bacillus cereus.* The 16S rRNA was used specifically because it is present in almost all bacteria, often existing as a multigene family or operons, so the bacterial strain would be identified correctly. Also, the function of the 16S rRNA gene over time has not changed, suggesting that no changes are affecting the identity of bacterial strains to report that 16S rRNA is random sequence a more accurate identity measure over time.

*B. cereus* and *B. thuringiensis* are closely related *Bacillus* species, but the microscopic examination showed that the selected strain did not produce any crystal toxins, to exclude the probability of *B. thuringiensis*, as well, this phylogenetic tree reports that the isolated *Bacillus* strain (SE42) is closer to *B. cereus* than *B. thuringiensis* with 99% of similarity with *B. Cereus* species as seen in bootstrap values (Baumann et al. 1984).

Regarding chitosanase activity to produce COSs, the highest chitosanase enzyme activity (1190 ± 20.1 U L^−1^) was observed at certain conditions (10 g colloidal chitosan, 0.5 g NaCl, 5 g yeast extract, 0.5 M KCl, 1 g NH4Cl, 0.24 g MgSO4, 0.7 g NaH2PO4, 0.34g Na2HPO4, 0.1 g CaCl2 and incubated at 30 °C for 48 h). Similarly, Choi et al. (2004), Liang et al. (2015), and Samrot et al. (2019) reported that the highest chitosanase production from *Bacillus* spp. was (1200 U L^−1^, 610 U L^−1^, and 82,000 U L^−1^, respectively). Also, Liaqat et al. (2018) and Cahyaningtyas et al. (2021) reported that the highest chitosanase activity was detected in *B. mojavensis* and *B. cereus* strains (2240 U L^−1^ and 500 U L^−1^, respectively).

A mixture of different molecular weights and DPs COSs oligomers was obtained in their pure form after size exclusion column chromatography. The purified COSs mixture was detected and confirmed as GlcN with different molecular weights and DPs using the UPLC and Mass spectra. These COSs’ molecular weights and DPs align with previous studies (Abassi et al. 2020; Kim et al. 2013; Purushotham and Podile 2012).

The results of in vivo toxicity assessment revealed that the lethal dose (LD_50_) in mice for COSs was above 1000 mg kg^−1^. Similar results were obtained from several studies (Fernandes et al. 2010; Punarvasu and Prashant 2023), which found that the administration of COSs at a concentration of 1000 mg kg^−1^ and above did not exhibit any toxicological symptoms, behavioral alterations, or fatality.

A marked immunostimulatory effect by COSs with different molecular weights was investigated as a result of elevation in some of the immunomodulatory cytokine markers such as IL-6, 10, and 12 and TNF-α (Zheng et al. 2016).

In this study, it has been observed that COSs have immunostimulatory effects on the cyclophosphamide-immunosuppressed mice, showing that the highest increase in spleen and thymus indices was among COSs-treated groups with 1000 µg kg^−1^ concentration (*p* < 0.001).

It agrees with Mei et al. (2013), who found that the spleen and thymus indices of the group administered COSs (1000 µg kg^−1^) were considerably higher when compared to the non-treated groups (*p* < 0.05 and < 0.01, respectively). These findings affirm that the high concentration of COSs can counteract the immunosuppressive effects of Cyclophosphamide.

In the thymus and spleen samples, the IL-6, TNF-α, and INF-γ levels were significantly (*p* < 0.01) higher in all COSs-treated groups than in cyclophosphamide-treated and control groups, specifically, the COSs-treated group with concentration 1000 µg kg^−1^ (*p* < 0.01). Similarly, Wu et al. (2015) found that COSs exhibited immunostimulatory activity, which was dose-dependent, as with higher doses significantly inducing the production of IFN-γ, IL-6, and TNF-α (*p* < 0.05), and Wei et al. (2009) found that the TNF-α and IFN-γ expression can be increased by different concentrations and molecular weights of COS (*P* < 0.01).

In addition, Yang et al. (2019) found that the TNF-α and IL-6 production were increased by COSs treatment in a dose-dependent manner. While Kim et al. (2006a, b) and Mei et al. (2013) reported that IFN-γ levels were markedly elevated in the COSs group compared to the controls (*P* < 0.05), the level of TNF-α was reduced following the COSs treatment.

The COSs were suggested to exhibit a potent anti-inflammatory effect due to their emerging activity against different inflammatory markers such as prostaglandins, cyclooxygenases, and histamines (Yang et al. 2016). Few in vivo studies focused on the anti-inflammatory effect of COSs, which are administered orally. During the anti-inflammatory effect evaluation by the right hind paw method, the elevation of edema 1 h after administering carrageenan is due to histamines, prostaglandins, and cyclooxygenase production (Lopes et al. 2020).

Dexamethasone, as an anti-COX-II inhibitor, showed a significant decrease in paw edema after 4 and 24 h, with a reduction in edema of 21.8% and 52.1%, respectively. The paw edema reduction was obtained after treatment with COSs at a concentration of 1000 mg kg^−1^ with 19.6% and 54.3%, after 4 and 24 h, respectively, as significantly (*p* < 0.01) the same effect as dexamethasone.

The anti-inflammatory effect of COSs (1000 mg kg^−1^) may result from interfering with cyclooxygenase pathways (de Andrade et al. 2021). After 48 h, it has been noted that there is no significant difference (*p* < 0.05) between COSs with concentrations of 500 and 1000 mg ml^−1^ and with dexamethasone (*p* < 0.05) to report that COSs have a similar anti-inflammatory activity to the potent anti-COX-II dexamethasone.

On the other hand, a low concentration of COSs (50 mg kg^−1^) was not enough to inhibit the hind paw edema because of its mild anti-inflammatory effects. The obtained result is in agreement with that reported by Fernandes et al. (2010) who investigated that after 48 h, COSs with both concentrations (1000 mg kg^−1^ and 500 mg kg^−1^) presented a similar effect on hind paw edema reduction (between 43 and 47%), with no significant difference to dexamethasone (*p* > 0.05). These findings can conclude that a COSs concentration of 500 mg kg^−1^ is a promising anti-inflammatory drug that can be used orally.

In conclusion, the tested COSs in this study showed promising and potential activities as an anti-inflammatory and immunostimulatory agent. These observed activities resulted from the ability of COSs to stimulate the production of different cytokine markers in safe doses.

## Data Availability

All data in this article were gathered, prepared, and analyzed during this study processing. The identified sequence of the locally isolated *Bacillus cereus* strain has been deposited in NCBI GenBank with accession no. PP093334.1. Also, the SE42 strain was added to the World Data Centre for Microorganisms (CCASU) (The strain number, CCASU-2024-71). All other data is available on a reasonable request.

## Abbreviations

ANOVA: Two-way analysis of variance
COSs: Chitooligosaccharides
DNS: Dinitrosalicylic acid
IL-6: Interleukin-6
INF-γ: Interferon gamma
RCMV: Regional Center for Mycology & Biotechnology
TNF-α: Tumor necrosis factor alpha
